# Automated quantification of PET/CT skeletal tumor burden in prostate cancer using artificial intelligence: The PET index

**DOI:** 10.1007/s00259-023-06108-4

**Published:** 2023-01-18

**Authors:** Sarah Lindgren Belal, Måns Larsson, Jorun Holm, Karen Middelbo Buch-Olsen, Jens Sörensen, Anders Bjartell, Lars Edenbrandt, Elin Trägårdh

**Affiliations:** 1 Division of Nuclear Medicine, Department of Translational Medicine, Lund University, Malmö, Sweden; 2 Department of Surgery, Skåne University Hospital, Malmö, Sweden; 3 Wallenberg Center for Molecular Medicine, Lund University, Malmö, Sweden; 4 Eigenvision AB, Malmö, Sweden; 5 Department of Nuclear Medicine, Odense University Hospital, Odense, Denmark; 6 Division of Radiology, Department of Surgical Sciences, Uppsala University, Uppsala, Sweden; 7 Division of Urological Cancer, Department of Translational Medicine, Lund University, Malmö, Sweden; 8 Department of Molecular and Clinical Medicine, Institute of Medicine, Sahlgrenska Academy at University of Gothenburg, Gothenburg, Sweden

**Keywords:** PET-CT, Artificial intelligence, Deep learning, Tumor burden, Prostate cancer

## Abstract

**Purpose:**

Consistent assessment of bone metastases is crucial for patient management and clinical trials in prostate cancer (PCa). We aimed to develop a fully automated convolutional neural network (CNN)-based model for calculating PET/CT skeletal tumor burden in patients with PCa.

**Methods:**

A total of 168 patients from three centers were divided into training, validation, and test groups. Manual annotations of skeletal lesions in [^18^F]fluoride PET/CT scans were used to train a CNN. The AI model was evaluated in 26 patients and compared to segmentations by physicians and to a SUV 15 threshold. PET index representing the percentage of skeletal volume taken up by lesions was estimated.

**Results:**

There was no case in which all readers agreed on prevalence of lesions that the AI model failed to detect. PET index by the AI model correlated moderately strong to physician PET index (mean *r* = 0.69). Threshold PET index correlated fairly with physician PET index (mean *r* = 0.49). The sensitivity for lesion detection was 65–76% for AI, 68–91% for physicians, and 44–51% for threshold depending on which physician was considered reference.

**Conclusion:**

It was possible to develop an AI-based model for automated assessment of PET/CT skeletal tumor burden. The model’s performance was superior to using a threshold and provides fully automated calculation of whole-body skeletal tumor burden. It could be further developed to apply to different radiotracers. Objective scan evaluation is a first step toward developing a PET/CT imaging biomarker for PCa skeletal metastases.

**Supplementary information:**

The online version contains supplementary material available at 10.1007/s00259-023-06108-4.

## Introduction

Accurate and consistent assessment of bone metastases is crucial for prognosis, treatment planning, and follow-up in patients with primary advanced or recurrent prostate cancer (PCa). Whole-body bone scan has been the most widely used modality to detect bone metastases. To overcome reader subjectivity, the automated bone scan index (aBSI),which reflects tumor burden as the fraction of the total skeleton weight, was developed [1]. A phase III randomized clinical trial showed that aBSI is an independent prognostic imaging biomarker for overall survival in metastatic castrate-resistant PCa [2]. The concept of aBSI demonstrates the strengths of extracting quantifiable information from an otherwise subjectively interpreted imaging study.

Advances toward developing more sensitive imaging techniques for diagnosing metastatic PCa include positron emission tomography combined with computed tomography (PET/CT) using, for example, prostate-specific membrane antigen (PSMA)-targeting radiopharmaceuticals or [^18^F]fluoride. A caveat, however, is that the interpretation depends on the individual reader’s training and experience. Definition of disease progression is especially problematic. Although recent criteria for more consistent reporting and definition of treatment response in PSMA PET/CT have been suggested, the central issue of reader subjectivity is still not fully eliminated [3–6].

Volumetric quantification of skeletal tumor burden in PET/CT has shown correlation to survival in patients with PCa [7–10], demonstrating its potential as a prognostic imaging biomarker similar to aBSI. Several methods have been proposed for quantification of whole-body skeletal tumor burden in [^18^F]fluoride PET/CT and whole-body tumor burden in PSMA PET/CT, most of them being semi-automated and often based on fixed standardized uptake value (SUV) thresholds [11–18]. The majority of these methods require drawing volumes of interest to encompass regions of suspicious uptake and manual exclusion of uptake unrelated to bone metastases, making them labor-intensive and subject to individual interpretation despite their semi-automated feature. However, recent leaps forward in methodology include fully automated convolutional neural network (CNN)-based quantification of whole-body tumor burden in PSMA PET/CT [19, 20].

A next step within this field would be to develop a fully automated method for assessment of PET/CT in metastatic PCa that can be reported consistently and quantitatively as an imaging biomarker. This is in line with the Prostate Cancer Working Group 3 recommendations that emphasize focusing on how biomarkers can be developed to predict outcome, guide management, and influence clinical decision-making [21]. Deep learning has become a dominant method in complex imaging analysis tasks. Compared to using SUV thresholds, CNNs trained on data from both PET and CT scans can handle more difficult uptake patterns and more accurately distinguish malignant foci from physiological uptake.

The primary aim of this study was to develop a completely automated CNN-based model for calculation of PET/CT whole-body skeletal tumor burden in patients with PCa, called the PET index, using [^18^F]fluoride PET/CT scans from three different hospitals. A secondary aim was to assess interreader agreement by comparing the PET index to manual interpretations by specialists in nuclear medicine and to a threshold-based model.

## Material and methods

### Patients

[^18^F]fluoride PET/CT scans from 90 patients at Skåne University Hospital, Sweden, 50 patients at Odense University Hospital, Denmark, and 34 patients from Uppsala University Hospital, Sweden, were screened retrospectively for eligibility. The scans were acquired as part of previous studies [22–24].

The previous inclusion criteria for the study at Skåne University Hospital were biopsy-verified high-risk PCa considered for curative treatment and a recent whole-body bone scan with normal or inconclusive findings [22]. The inclusion criteria for the study at Odense University Hospital were biopsy-verified PCa and a whole-body bone scan with ≥ 1 bone metastasis [23]. The inclusion criteria for the study at Uppsala University Hospital were biopsy-proven PCa and Gleason score ≥ 8 [24].

Of the 174 patients, three were excluded from the current study due to extremely high metastatic skeletal burden with confluent uptake, disabling marking of individual foci. Additionally, three patients were excluded due to inaccurate SUVs as a consequence of incorrect image labeling. In total, 168 patients were included in the final analysis. The study was approved by the regional ethical review board in Lund (2016/443) and Uppsala (2012/347), Sweden, and by the Danish Patient Safety Authority (3–3013-1692/1).

### PET/CT imaging

At Skåne University Hospital, PET/CT scans were acquired with Gemini TF (Philips Medical Systems) 1–1.5 h after i.v. injection of 4 MBq/kg (max dose 400 MBq) of [^18^F]fluoride, with 2 min per bed position. The PET images were reconstructed using the BLOB-OS-TOF algorithm, with 3 iterations, 33 subsets, to a 144 × 144 matrix with a pixel size and slice thickness of 4 mm. A low-dose CT (Smart mA, 120 kV, 30–160 mA, slice thickness 5 mm) was obtained for attenuation correction and image fusion. A diagnostic CT with i.v. and per oral contrast was performed 1–24 days previously as part of another PET/CT scan within the previous study frame. The scans were acquired from the vertex to the mid thigh.

PET/CT scans at Odense University Hospital were obtained by Discovery VCT 64 (GE Healthcare)1 h after i.v. injection of 3 MBq/kg of [^18^F]fluoride, with 2.5 min per bed position. The PET data was reconstructed with a matrix size of 128 × 128, pixel size 5.47 mm, and a slice thickness of 3.27 mm using OSEM (2 iterations, 28 subsets). A low-dose CT was acquired using tube current modulation (SmartmA, 140 kV, 30–150 mA) and was reconstructed in a field of view of 50 cm using filtered back projection, slice thickness of 3.75 mm, and spacing of 3.27 mm. Scans were acquired from the base of the skull to the mid thigh.

At Uppsala University Hospital, PET/CT scans were acquired with Discovery ST (GE Healthcare) 1 h after i.v. injection of 3 MBq/kg body weight of [^18^F]fluoride, with 2 min per bed position. Images were reconstructed using OSEM (2 iterations, 21 subsets), a matrix size of 128 × 128, pixel size 3.9 mm, and slice thickness of 3.27 mm. A low-dose CT was performed immediately before the PET (140 kV, 10–80 mA, slice thickness 3.75). Scans were acquired from the vertex to the proximal 1/3 of the femur.

### Training and validation groups

For training, 53 patients from Skåne University Hospital and 29 patients from Odense University Hospital (constituting approximately 60% of the entire number of patients from each center) were randomly selected. The 34 scans from Uppsala University Hospital did not have any pathological uptake and were added to improve the network’s ability to handle non-suspicious uptake regions. A total of 116 patients were used for training.

Manual segmentations of suspicious skeletal uptake were performed using a cloud-based segmentation tool (www.recomia.org) [25]. A single physician segmented all uptakes with malignant or unclear origin based on the original clinical written reports. The segmentations were visually re-examined by a specialist in nuclear medicine with 7 years of PET/CT experience before the final training.

The model was evaluated in a validation group with 26 annotated PET/CT scans (18 from Skåne University Hospital and eight from Odense University Hospital), constituting approximately 20% of patients from each of the two centers.

### Test group

The test group consisted of 26 patients (18 from Skåne University Hospital and eight from Odense University Hospital). Three specialists in nuclear medicine from one center (readers A–C) with 7–10 years of experience each performed manual segmentations of lesions in the PET/CT scans in the test group. A physician from another center who had segmented the training material (reader D) also performed manual segmentations of suspicious uptake in the test group patients in order to investigate any bias for the AI model toward this reader. The same tool was used for the training material [25]. The readers performed the segmentations separately and individually. Readers A–C were blinded to all patient information. Reader D had access to patient characteristics and original clinical written reports from the PET/CT scans. The readers were instructed to label all voxels judged to correspond to malignant uptake in any of the three planes (axial, coronal, or sagittal) in each slice. Uptake of unclear origin where metastasis could not be excluded was instructed to be labeled as malignant. The manual segmentation of each lesion was based on information from both the PET and CT scans. Written guidelines were established (Supplementary material 1) and accepted by all the readers before the segmentation started.

A threshold of SUV 15 has been used in previous studies to automatically segment lesions in [^18^F]fluoride PET/CT [14, 26]. We employed this threshold to our test group in order to compare to the AI model. Segmented connected components < 0.1 ml were removed.

### AI model

**Fig. 1:**
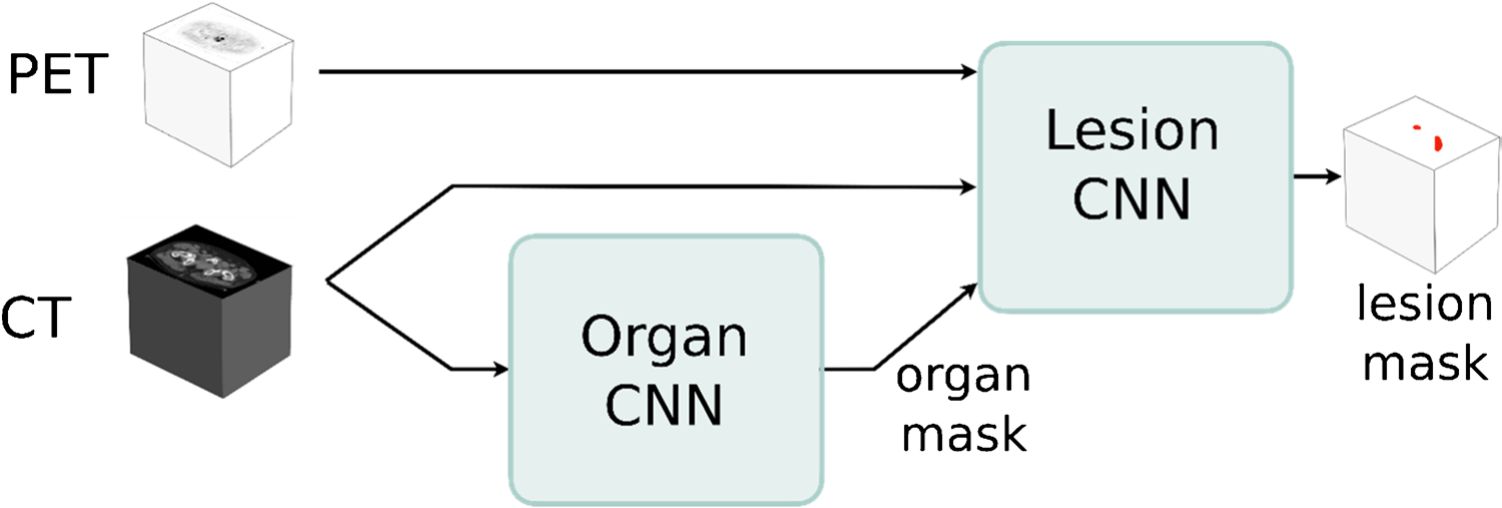
Schematic of the AI model. The lesion CNN segments suspected metastatic skeletal uptake in the scan with a CT, PET scan, and a label mask produced by the organ CNN as input

The CNN-based segmentation model consists of two fully convolutional networks, the organ CNN and the lesion CNN. The organ CNN segments 100 different organs (77 bones and 23 soft tissue organs) using a CT image as input [25]. The output from the organ CNN is used to create a label mask marking bones, joints, kidneys, lungs, brain, skull, spleen, heart, aorta, and liver. This mask, as well as the CT and PET images, is used as input for the lesion CNN that classifies each voxel as either bone metastasis or background (Fig. 1). The label mask is added as input to facilitate the differentiation between malign and benign high uptake regions. Hence, the organs marked are either organs where malignant uptake or physiological high uptake is common.

Both CNNs have the same structure as 3D U-Net [27]. The input of the lesion CNN is a 100 × 100 × 100 volume of voxels where each voxel has a size of 3.0 × 1.37 × 1.37 mm. The different input modalities (CT, PET, and label mask) are resampled using trilinear interpolation and concatenated across the feature or channel, dimension. For this input size, the CNN outputs an estimated class probability for each voxel of a 12 × 12 × 12 volume at the center of the input patch. Training was performed with categorical cross-entropy as loss and training samples were drawn evenly from the two classes. In addition, training samples were drawn more often from areas with high SUV uptake and voxels misclassified by earlier versions of the CNN. This is to focus the training on “harder” samples since many areas, especially those with low uptake, are easily classified as background.

During inference, the lesion CNN was applied to all voxels in the image. As a postprocessing step, all connected components < 0.1 ml were removed. In addition, all areas where high uptake regions were deemed to originate from joints were removed. More specifically, each voxel was associated with local maxima in the SUV image using a watershed transform. Voxels where these local maxima were located in a joint were set to background. The joint mask was created using bone segmentations output from the organ CNN.

### PET/CT quantitative analysis

To calculate the PET index, the volume of all tracer uptake marked as suspicious by each reader/model is summed and divided by the total volume of visualized skeletal parts in each patient, generating a measure of skeletal tumor burden expressed in percent. The bone volume was calculated using a previously described CNN-based method [28].

### Statistics

Patient characteristics (age and PSA levels) for the training, validation, and test groups were compared using Kruskal–Wallis H test. A *p*-value < 0.05 was considered a statistically significant difference.

Bland–Altman plots were used to assess interreader reliability in PET index estimation between the readers and the AI model. The correlation between PET indices calculated by each reader, the AI model, and the threshold model in each patient was assessed using Spearman rank correlation. Correlation was considered very strong for Spearman’s coefficient *r* absolute values > 0.8, moderately strong for *r* = 0.6–0.79, fair for *r* = 0.3–0.59, and poor for *r* < 0.3 [29].

For analysis on both patient and lesion-level, each of the four readers was alternately held as reference and pairwise compared to another reader, AI, or threshold model [30]. The pairwise results were then averaged to obtain results for reader vs. reader, AI model vs. reader, and threshold model vs. reader.

For patient-level analysis, a true positive (TP) scan prediction was defined as the detection of suspicious uptake corresponding to a PET index > 0 in a scan where a reader used as reference also segmented suspicious uptake defined as a PET index > 0. Otherwise, it was considered false negative (FN). A false positive (FP) scan prediction for reader or model was defined as a PET index > 0 in the same scan where a reader used as reference did not identify suspicious uptake (PET index = 0). The sensitivity and positive predictive value (PPV) was calculated based on these values for reader vs. reader, AI model vs. reader, and threshold model vs. reader.

For lesion-level analysis, lesions were defined as TP for reader, AI, or threshold model, respectively, in case of either full or partial segmentation overlap with a reader held as reference, or else, they were defined as FN. FP lesions were defined as zero segmentation overlap by reader or model, with a reader held as reference. The sensitivity for the detection of lesions was calculated as the percentage of detected lesions by each individual reader, also detected by a reader or a model. The PPV was evaluated as the percent of TP lesions for a reader or a model when compared to another reader used as reference, divided by TP plus FP lesions when compared to the same reference reader. Data were analyzed using IBM SPSS Statistics 28.

## Results

### Patient characteristics

Information regarding age, PSA levels, and Gleason scores for the patients in the training, validation, and test groups is presented in Table 1. There was no statistically significant difference between age and PSA levels when comparing the training, validation, and test groups (*p* > 0.05).

**Table 1:** Patient characteristics. Information regarding age, PSA levels, and Gleason score was unavailable for the 34 patients in the training group that were added to increase the model’s ability to handle non-suspicious high uptake regions

Median (IQR)	Training group (*n* = 82)	Validation group (*n* = 26)	Test group (*n* = 26)	*p*-value
Age, years	68 (64–72)	69 (64–73)	66 (64–72)	0.479
PSA, ng/mL	30 (16–77)	29 (19–57)	28 (14–38)	0.626
Gleason score	8 (7–9)	8 (7–9)	8 (7–9)	

### PET index

PET indices calculated by readers A–D, the AI model, and the threshold model are presented in Table 2.

**Table 2:** Mean and standard deviation (SD), as well as median and interquartile range (IQR) PET indices for the patients in the test group calculated by the readers and AI model and by using a threshold of SUV 15 (*n* = 26)

	PET index, %					
	Reader A	Reader B	Reader C	Reader D	AI model	Threshold model
Mean (SD)	0.70 (1.68)	0.91 (2.20)	0.73 (1.86)	1.35 (1.51)	1.44 (3.27)	1.35 (3.16)
Median (IQR)	0 (0–0.96)	0 (0–0.04)	0 (0–0.06)	0.01 (0–0.14)	0.02 (0–0.96)	0.03 (0–0.41)

### Patient-level analysis

**Fig. 2:**
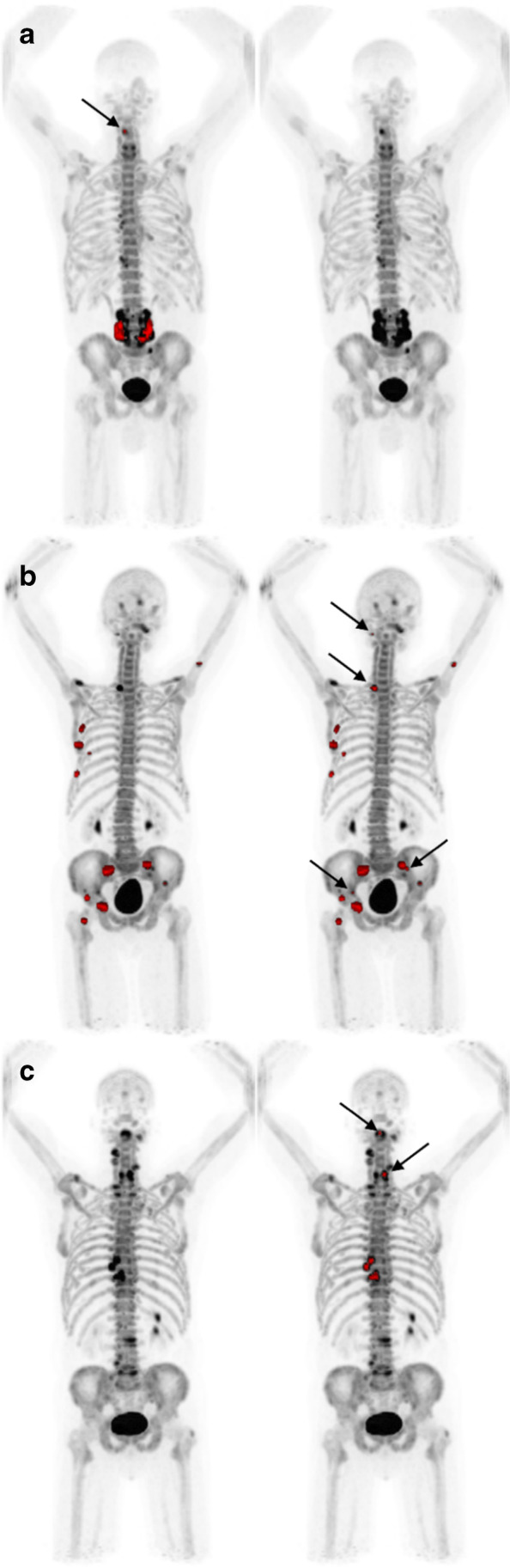
**a** Left: the AI model detects suspected a metastatic uptake in the lumbar spine and a small lesion in the cervical spine (arrow), marked in red. PET index 0.57%. Right: none of the physicians identify any lesions in the same patient. Of note, the patient had previously undergone lumbar spinal fusion. **b** Left: the AI model identifies several foci in the right ribcage, pelvis, and right hip. PET index 1.34%. Right: reader D identifies additional lesions in the skull, spine, and pelvis (arrows). PET index 1.42%. **c** Left: the AI model and the readers do not detect any lesions. Right: the threshold marks several lesions in the cervical (arrows) and thoracic spine. PET index 0.16%

On a patient-level, readers A–D were unanimous in 73% (19/26) of patients in the test group regarding prevalence (*n* = 12) and absence (*n* = 7) of suspected metastatic uptake. There was no case in which all readers agreed on the prevalence of lesions that the AI model failed to detect. In six patients, none of the readers identified any lesions, while the AI model found lesions corresponding to PET indices up to 0.57% (Fig. 2a). A visual examination showed that lesions detected by the AI model but not by the readers were most often located in the spine or costovertebral joints. When comparing the AI model to reader D from a different center who had performed the manual segmentations used for training, interpretation regarding prevalence/absence of metastatic uptake coincided in 62% (16/26) of the patients (Fig. 2b).

The threshold model identified lesions corresponding to PET indices up to 0.25% in nine patients, while all readers agreed on the absence of lesions (Fig. 2c). In one patient, the threshold did not detect any lesions, while all readers and the AI model agreed on suspicious uptake in the right ribcage.

**Fig. 3:**
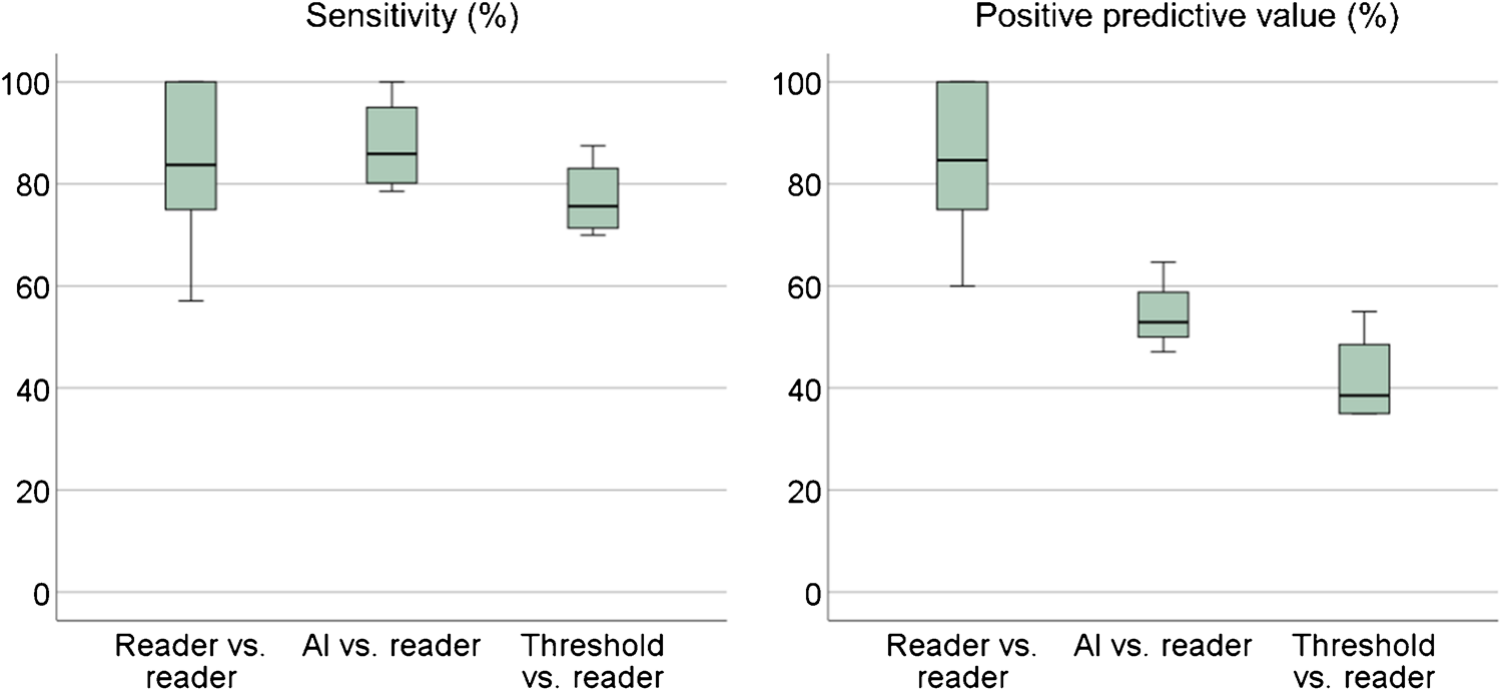
Boxplots of the sensitivity and positive predictive value for the detection of suspicious uptake at a patient-level for reader vs. reader, AI model vs. reader, and threshold model vs. reader. One of readers A–D was alternately held as reference and pairwise compared to another reader or model

Boxplots of the sensitivity and PPV for the detection of lesions on a patient-level are shown in Fig. 3. The median (range) sensitivity for reader vs. reader, AI model vs. reader, and threshold model vs. reader was 83.8% (57.1–100%), 85.9% (78.6–100%), and 75.6% (70.0–87.5%), respectively, depending on which reader was used as a reference. The median (range) PPV for reader vs. reader, AI model vs. reader, and threshold model vs. reader was 84.7% (60.0–100%), 52.9% (47.1–64.7%), and 38.6% (35.0–55.0%), respectively.

**Fig. 4:**
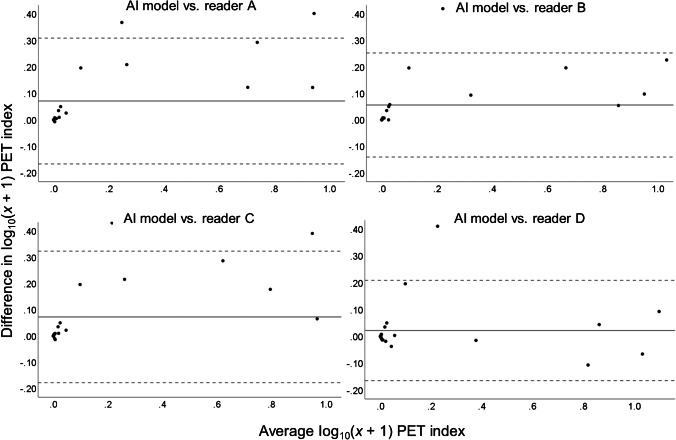
Bland–Altman plots illustrating the interreader reliability between the AI model and each physician regarding PET index estimation after log_10_(*x* + 1) transformation (*n* = 26). The solid line represents mean differences between the two log_10_(*x* + 1)-transformed PET indices. The dotted lines indicate the upper and lower 95% limits of agreement (mean difference ± 1.96 multiplied by the standard deviation of the mean difference). The same plots with subject labeling are provided in Supplementary material 2. The seven data points outside the most-leftward main cluster in all four plots correspond to the same subjects

Bland–Altman plots illustrating interreader reliability between the AI model and each reader for log_10_(*x* + 1)-transformed data are displayed in Fig. 4. The back-transformed limits of agreement were 4.3 (upper) and − 2.8 (lower) for AI vs. reader A, 2.9 (upper) and − 1.9 (lower) for AI vs. reader B, 4.1 (upper) and − 2.7 (lower) for AI vs. reader C, and 4.4 (upper) and − 2.8 (lower) for AI vs. reader D. With an increasing mean PET index, the difference between reader and AI model estimation tended to increase for all the four readers. A visual examination of the scans with the highest PET index discrepancies revealed that segmentations of the same lesions were often performed but with larger individual areas marked by the AI model and/or segmentation of fractures or more uncommon uptake patterns (such as subject number 26, Fig. 2a).

**Fig. 5:**
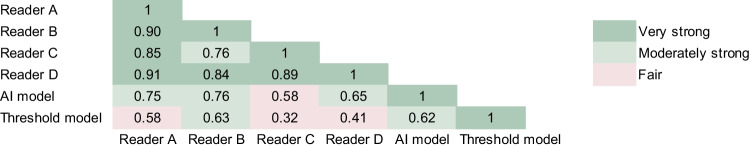
Spearman correlation coefficients (*r*) between PET index calculated by the readers, AI model, and SUV 15 threshold model for the patients in the test group (*n* = 26). Correlation is considered very strong for *r* > 0.8, moderately strong for *r* = 0.6–0.79, fair for *r* = 0.3–0.59, and poor for *r* < 0.3

PET indices calculated by the AI model showed a fair-moderately strong correlation to reader PET indices in the same patient (Fig. 5). Pairwise interreader correlation between readers A–D was moderately-very strong. PET indices calculated by using a threshold of SUV 15 correlated fairly with all the physicians and the AI model.

### Lesion-level analysis

Table 3 shows the number of TP, FP, FN, sensitivity, and PPV for readers A–D, the AI model, and the threshold model. The detection sensitivity for suspicious lesions when considering each reader as reference ranged between 68 and 91% depending on which reader was used as a reference. The sensitivity of the AI model ranged from 65 to 76% when considering each reader as ground truth, respectively. The sensitivity of the threshold model when considering each reader as ground truth was 44–51% and thus outside of the interreader range. When comparing the AI model to reader D who had segmented the training material, the number of TP was 130 (5.0 per patient), FP 38.0 (1.5 per patient), and FN 61.0 (2.3 per patient), with a sensitivity of 68% and PPV of 77%.

**Table 3:** Mean (range) true positive, false positive, and false negative values, as well as sensitivity and positive predictive value (PPV), when using one of readers A–D alternately as a reference for pairwise comparison with another reader or the AI or threshold model, respectively. The results are than averaged over the choices of reference. The results are presented for the entire test group and per patient (*n* = 26)

	Reader vs. reader	AI model vs. reader	Threshold model vs. reader
True positives			
Total	133.8 (116.0–154.0)	122.5 (112.0–130.0)	81.8 (76.0–87.0)
Per patient	5.1 (4.5–5.9)	4.7 (4.3–5.0)	3.1 (2.9–3.3)
False positives			
Total	40.0 (14.0–62.0)	68.0 (38.0–123.0)	61.8 (57.0–65.0)
Per patient	1.5 (0.5–2.4)	2.6 (1.5–4.7)	2.4 (2.2–2.5)
False negatives			
Total	40.0 (14.0–62.0)	51.3 (37.0–67.0)	92.0 (73.0–107.0)
Per patient	1.5 (0.5–2.4)	2.0 (1.4–2.6)	3.5 (2.8–4.1)
Sensitivity (%)	77.5 (67.7–90.6)	71.0 (65.1–75.5)	47.3 (44.0–51.0)
PPV (%)	77.5 (67.4–90.8)	65.9 (50.0–77.4)	57.0 (53.9–59.6)

## Discussion

The results show that an AI-based model can be trained to automatically identify lesions in PET/CT to give a measure of the total skeletal tumor burden. This measure, called the PET index, is a fully automated model for calculation of whole-body skeletal tumor burden. Objective scan evaluation is a first step toward developing a PET/CT imaging biomarker for PCa skeletal metastases.

To qualify as decision support, it is important for an automated method not to dismiss a potentially metastatic uptake. This is important from a patient perspective as it will misjudge prognosis and lead to incorrect treatment. At the same time, identification of a large number of false positive lesions makes the interpretation time consuming, defeating its purpose. The AI model did not rule out metastatic uptake in any patient where the readers agreed on prevalence. The relative low number of false negatives came at the expense of higher false positive rate. At a patient-level, the sensitivity of the AI model was comparable to the readers, but the median PPV for the AI model was only 52.9%. In the clinical setting, these false positives scans can be readily disregarded by a reader as they often show a single uptake in the spine or a joint due to degenerative disease. At a lesion-level, the average number of false positives per patient was higher for the AI model (2.6) compared to the readers (1.5) but still low compared to another recent study in which the average number of false positive bone lesions in PSMA PET/CT was 8.3 per patient for an AI-based software [31]. These results are encouraging and with a larger training material, the performance of the model would most likely increase. Additionally, it remains important to have a nuclear medicine physician read the PET/CT scans to assess where the lesions are located to help guide management and to rule out uptake of non-malignant origin.

As the PET index represents the sum of all voxels marked as suspicious, segmentation of the same lesions may result in different PET indices depending on interreader differences in border assessment. PET index calculated by the AI model was generally higher compared to the readers, which is probably a reflection of the AI model’s propensity to segment individual lesions as larger volumes and/or due to the higher number of false positives. In patients with a high number of metastases, this effect is larger, as reflected in the increasing variability of the differences with the PET index magnitude. As PET index also depends on bone volume, it would be beneficial to standardize PET/CT scanning to consistently include the same parts of the humeri, femura, and skull in the field of view, which was not always the case in the scans included in this study. Similar to aBSI, inclusion of the skeletal volume in an indexed measure of tumor burden enables interpatient comparison.

At a patient-level, the readers agreed on occurrence of suspect metastatic uptake in < 3/4 of the patients. This shows that even among experienced specialists with the majority working at the same center, interpretation of PET/CT is subjective. One can speculate that agreement would most likely have been even lower had the study included readers from more centers and/or with greater discrepancies in experience. The visual inspection following the final analysis showed a misalignment between PET and CT scans in several patients, which is also very likely a contributing factor to the relatively low interreader agreement. The results suggest that there is an unmet need for objective and reproducible analysis of PET/CT. AI presents an interesting opportunity to meet this need.

A threshold of SUV 15 has been used in previous studies for segmentation of metastatic uptake in [^18^F]fluoride PET/CT [14, 26]. When applying this threshold to our test group, agreement regarding prevalence/absence of suspicious uptake at a patient-level and correlation between PET indices was considerably lower compared to the AI model using each physician as a reference. The threshold identified suspicious uptake in the majority of patients that the readers agreed did not have metastatic disease, and failed to identify suspicious uptake in one patient where all readers agreed on prevalence. The use of a fixed SUV threshold for segmentation of suspected metastatic uptake is problematic since [^18^F]fluoride activity in benign processes and malignant disease is known to overlap. Normal [^18^F]fluoride uptake also varies between different bone regions [32]. Our results support the application of AI to more accurately calculate skeletal tumor burden compared to applying a global SUV threshold.

In the clinical setting, one potential role for PET index is as an objective, quantitative measure of progression and response in bone during systemic treatment of metastatic PCa. Especially for [^177^Lu]Lu-PSMA-617 and analogues therapy, longitudinal assessment of changes in the tumor volume in PSMA PET/CT may be clinically useful [33, 34]. Quantitative assessment of tumor burden in patients scheduled for PSMA-directed radioligand therapies could also be used for personalized dosimetry. Further, prognostic information may be obtained through the assessment of tumor burden in patients with advanced PCa [10, 20, 34]. An AI-based model for quantification of tumor burden in PET/CT has a potential to risk-stratify patients with metastatic PCa, and to provide potentially prognostic and predictive information in the clinic. As exemplified by the development of aBSI, extensive analytical and clinical validation would be required. Ultimately, the correlation of an AI-based measure of tumor burden in PET/CT to patient outcome would have to be assessed in prospective, multicenter trials. In order to achieve general applicability, the model would profit from being further trained on a larger material containing patients from several centers with annotations performed by more than one reader, and to be evaluated in different patient cohorts. A future prospective is also to incorporate this model into an expanded one for detection and quantification of lymph node metastases as well as primary tumors.

A limitation of this study was the relatively small sample size used for training and testing. Because metastatic border ground truth is theoretically given by histopathology but is practically and ethically impossible to obtain, manual segmentations were used as the reference standard, which is also a limitation. However, the PET/CT scans used for training came from three different centers in two different countries, acquired using different cameras and imaging protocols. The combination of three centers in this development enhances the potential of the model to be applied to more than one setting and to facilitate its clinical translation. This, combined with using PET/CT scans from patients without metastatic bone disease but with the same characteristics as the test group, may have helped the training and potentially reduced the number of false positives.

Another limitation is that the study is based on a tracer which only measures skeletal and not all tumor burden. Although [^18^F]fluoride is recommended for bone imaging by several guidelines and is more sensitive in diagnosing skeletal metastases compared to conventional imaging [21, 35, 36], PSMA-targeting radiopharmaceuticals are the most promising tracers in PCa PET imaging. Compared to [^18^F]fluoride, they have the additional beneficial ability to diagnose extra-skeletal disease. In a recent study, the performance of a CNN-based model for identification and location classification of suspicious uptake in [^68^Ga]Ga-PSMA-11 PET/CT improved when 2-deoxy-2-[^18^F]fluoro-D-glucose PET/CT training data was added, so called ‘dual tracer learning’ [37]. The authors suggest that combining information from multiple tracers in network training is a promising method, considering the limited availability of expert-annotated PET/CT data. The work presented in this study therefore extends beyond [^18^F]fluoride PET/CT application and lies the foundation to developing a PSMA PET/CT imaging biomarker, for example, by pre-training a model on [^18^F]fluoride data with fine-tuning on PSMA scans. A next step would be to extend the PCa lesion detection and segmentation to encompass primary tumor, local spread, and distant disease to give a comprehensive measure of the tumor load. Automated identification and quantification of whole-body PSMA uptake could help establish its role in therapy response monitoring in metastatic PCa, which is currently a challenging and relatively underexplored scenario.

## Conclusions

We have introduced a fully automated AI tool for completely automated calculation of skeletal tumor burden in PET/CT. Diagnosing and tracking metastatic progression in an objective manner provided by AI support could be used in clinical trials for eligibility, risk stratification and evaluation of treatment effect. In a clinical setting, it can be used as decision support to expedite and standardize scan evaluation, and to evaluate bone response during systemic treatment. However, its full clinical value needs to be investigated in future studies evaluating patient outcome.

## Supplementary information

Below is the link to the electronic supplementary material.

Supplementary file1 (PDF 321 KB )

## Data Availability

The AI method is freely available for research at www.recomia.org.
