# Supplementary material for: Automated quantification of PET/CT skeletal tumor burden in prostate cancer using artificial intelligence: The PET index
Source: Eur J Nucl Med Mol Imaging. 2023 Jan 18;50(5):1510–20. doi: 10.1007/s00259-023-06108-4 (PMC10027829; doi:10.1007/s00259-023-06108-4)

Supplementary material

Supplementary material 1. Guidelines used by the readers for evaluation and segmentation of suspicious uptake in [^{18}F]fluoride PET/CT using the freely available AI tool RECOMIA (www.recomia.org).

Guidelines CT evaluation

- Articular charges are often considered benign
- Subcondral cysts with peripheral sclerosis are typically considered benign
- Exostoses/randosteophytes are considered benign
- Osteosclerotic foci with activity uptake are generally suspicious
- In the cervical column, care should be taken to appoint osteosclerotic foci, especially if they are transverse
- Osteolysis with bone activity is suspicious
- Vertebral collapse cannot really be judged whether it is benign or malignant, the same with fractures
- Schmorl's nodes are known for their distinctive appearance in sagittal sections
- Costal foci are more suspicious if elongated than if they are point-shaped
- The total amount of changes and their distribution are taken into account - for example, a solitary metastasis in the cervical column is unlikely, and successive costal foci suggest trauma rather than malignancy
- Obvious benign fractures, for example with continuous fracture line and even with callus formation, for example in costa, are considered benign even if solitary
- In the calvaria you have to be aware that it is not just calcifications in the meninges that can be observed
- Changes with a benign appearance on CT, for example rounded and well-defined sclerosis, such as a bone island, or for example bone changes that resemble a hemangioma, are considered benign even if there is a corresponding small focal uptake in the PET scan

Guidelines PET evaluation

- Classification of what represents increased radiotracer uptake due to metastatic disease should be based on your own individual clinical evaluation without consulting each other or another colleague
- Segmentations should be drawn individually and separately without consulting each other or another colleague

- Non-metastatic increased uptake judged to be caused by for example degeneration, traumatic fractures or prostheses/foreign material should not be marked
- Lesions which cannot be categorized as either clearly benign or malignant according to the before listed criteria, should be categorized as malignant
- The uptake should be marked in its entirety and its outlining should be drawn as accurately as possible based on what you judge to be the border. Hence, all voxels judged to correspond to metastatic uptake in any of the three planes (axial, coronal or sagittal) should be marked as metastasis in each slice

Supplementary material 2. The Bland-Altman analyses from Fig. 4 with numbers in the plots representing subject identification. The solid line represents mean differences between the two $\log_{10}(x + 1)$-transformed PET indices. The dotted lines indicate the upper and lower 95% limits of agreement.

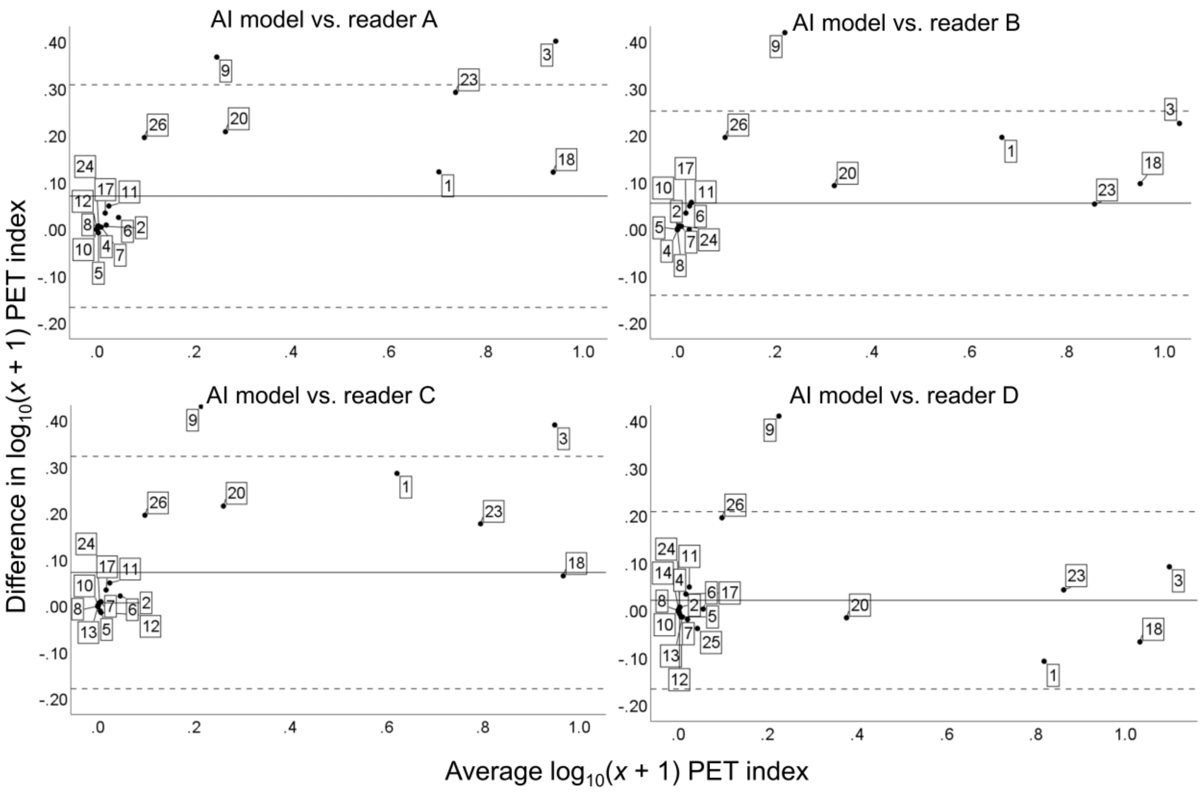

Supplement: Supplementary file 1 — Supplementary file1 (PDF 321 KB ) [file 259_2023_6108_MOESM1_ESM.pdf]
